# Contrasting Outcomes in Heyde Syndrome: Diffuse Small-Bowel Angiodysplasia Versus Treatable Colonic Angiodysplasia

**DOI:** 10.7759/cureus.114244

**Published:** 2026-08-10

**Authors:** Jihane Rizkou, Hiba Rizkou, Fatimaezzahra Lairani, Khadija Krati

**Affiliations:** 1 Gastroenterology and Hepatology, Mohammed VI University Hospital Center, Marrakech, MAR; 2 Cardiology, Mohammed VI University Hospital Center, Marrakech, MAR

**Keywords:** acquired von willebrand syndrome, angiodysplasia, aortic stenosis, argon plasma coagulation, capsule endoscopy, gastrointestinal bleeding, heyde syndrome

## Abstract

Heyde syndrome is characterized by the association of aortic stenosis, gastrointestinal bleeding from angiodysplasia, and acquired von Willebrand syndrome. We report two contrasting cases illustrating how lesion distribution and therapeutic accessibility may influence immediate bleeding management options and clinical course. The first patient was a 72-year-old male with severe calcific aortic stenosis who presented with recurrent melena and severe anemia. Upper gastrointestinal endoscopy and colonoscopy were negative, whereas capsule endoscopy revealed numerous angiodysplastic lesions diffusely distributed throughout the small bowel. Transthoracic echocardiography showed an aortic valve area of 0.72-0.74 cm², a mean transvalvular gradient of 43.40-45.16 mmHg, a peak aortic jet velocity of 4.02-4.04 m/s, and a left ventricular ejection fraction of 55%. Von Willebrand factor activity was reduced, with an activity-to-antigen ratio of 0.41. Device-assisted enteroscopy was not available at our center, limiting endoscopic therapeutic options. Despite transfusion and supportive management, persistent gastrointestinal bleeding progressed to hemorrhagic shock, and the patient died before definitive aortic valve intervention could be performed. The second patient was a 70-year-old male with severe calcific aortic stenosis who presented with recurrent hematochezia. Colonoscopy identified a localized right-colonic angiodysplasia that was successfully treated with argon plasma coagulation. Echocardiography showed an aortic valve area of 0.60 cm², a mean transvalvular gradient of 54.18 mmHg, a peak aortic jet velocity of 4.61 m/s, and a left ventricular ejection fraction of 65%. The von Willebrand factor activity-to-antigen ratio was 0.46. No recurrent gastrointestinal bleeding was documented during one year of follow-up after endoscopic treatment; however, completion of the planned aortic valve intervention could not be confirmed from the available records. These cases emphasize the importance of early recognition, lesion localization, and timely multidisciplinary management while illustrating that clinical outcome in Heyde syndrome is multifactorial.

## Introduction

Heyde syndrome is defined by the association of aortic stenosis, gastrointestinal bleeding from angiodysplasia, and acquired von Willebrand syndrome [1-4]. Since the initial observation reported by Heyde in 1958, clinical and mechanistic studies have supported an association between severe aortic stenosis and recurrent gastrointestinal bleeding [1-3,5-8].

The principal pathophysiological mechanism involves increased shear stress across the stenotic aortic valve. This stress induces conformational changes in circulating von Willebrand factor (vWF), exposing cleavage sites and promoting proteolysis of its high-molecular-weight multimers by ADAMTS13 [3,4]. The resulting qualitative hemostatic defect impairs platelet adhesion and increases the tendency to bleed from gastrointestinal vascular lesions such as angiodysplasias [5-7].

In clinical practice, acquired von Willebrand syndrome may be supported by a disproportionate reduction in vWF activity relative to antigen concentration, whereas demonstration of the loss of high-molecular-weight multimers provides more specific laboratory confirmation when multimer analysis is available [3,4]. Correction of severe aortic stenosis by surgical or transcatheter aortic valve replacement is considered central to definitive management of Heyde syndrome [7].

Angiodysplasias may involve the colon or small bowel, and their distribution has important diagnostic and therapeutic implications. Localized colonic lesions may be accessible to conventional endoscopic hemostasis, whereas multiple small-bowel lesions may require capsule endoscopy for diagnosis and device-assisted enteroscopy for treatment [9-12]. We report two contrasting cases of Heyde syndrome illustrating how lesion localization and therapeutic accessibility may influence immediate management while highlighting the multifactorial nature of clinical outcome.

## Case presentation

Case 1

A 72-year-old male was admitted for recurrent melena that had been evolving for approximately one month, associated with progressive fatigue, asthenia, and symptomatic anemia. His medical history included severe calcific aortic stenosis under cardiology follow-up and arterial hypertension. He was not receiving anticoagulant or antiplatelet therapy and had no previous history of gastrointestinal bleeding.

On admission, the patient was pale but hemodynamically stable. Cardiovascular examination revealed a systolic ejection murmur predominantly heard over the aortic area. There were no clinical signs of congestive heart failure, including peripheral edema or pulmonary crackles. Laboratory investigations demonstrated anemia, with a hemoglobin level of 8.3 g/dL, thrombocytopenia with a platelet count of 112,000/mm³, and a markedly elevated C-reactive protein level of 157 mg/L. Renal function was preserved.

Given the marked inflammatory response and thrombocytopenia, a competing infectious process was considered. The patient was afebrile, blood cultures were negative, and echocardiography demonstrated no evidence of valvular vegetation. No alternative infectious or malignant cause was identified during the available evaluation.

**Figure 1:**
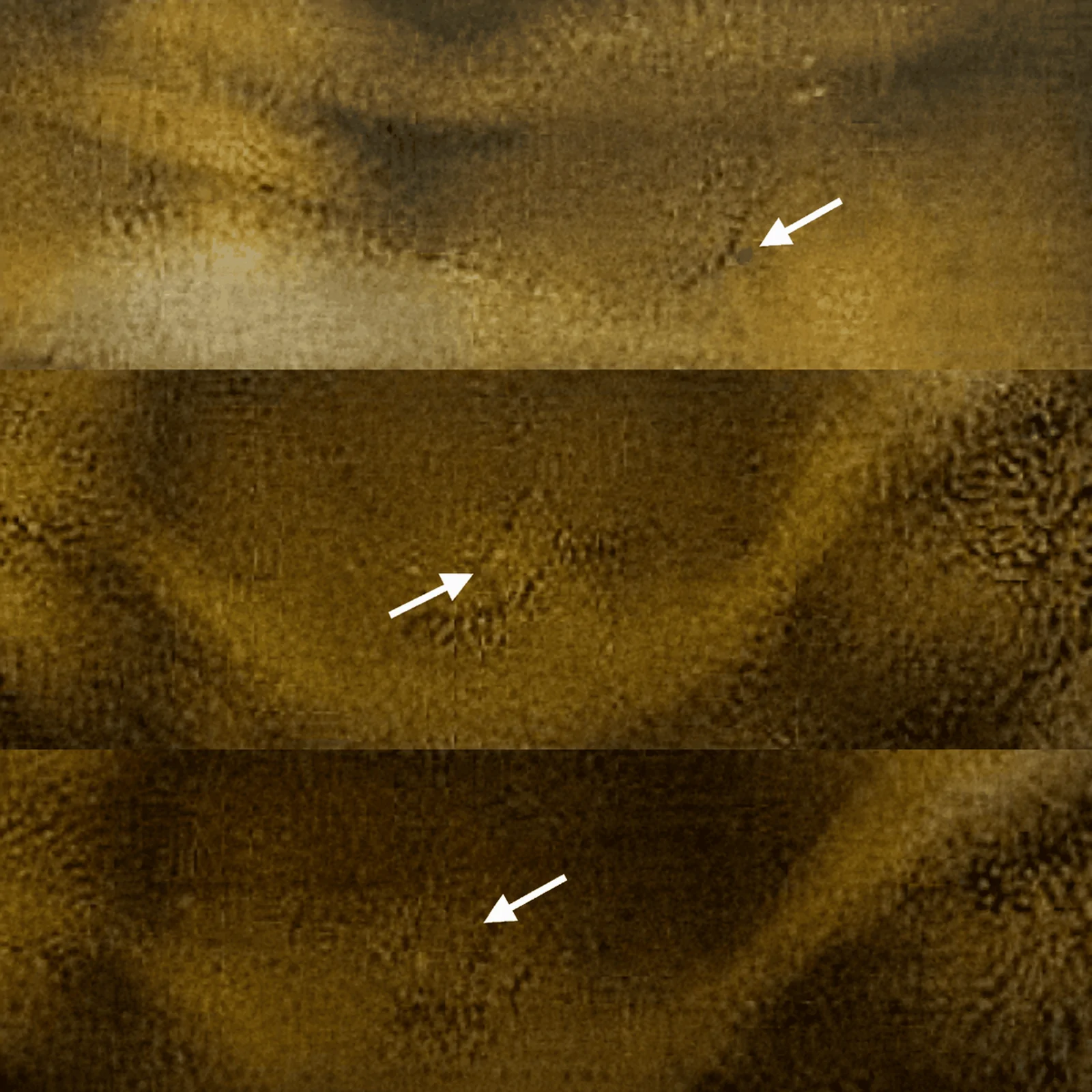
Capsule endoscopy findings in Case 1. Representative capsule endoscopy frames showing multiple small-bowel vascular ectasias consistent with angiodysplasia. White arrows indicate representative lesions. Numerous similar lesions were diffusely distributed throughout the small bowel, precluding a reliable numerical count.

Upper gastrointestinal endoscopy and colonoscopy were initially performed but did not identify the bleeding source. Because of persistent overt gastrointestinal bleeding, capsule endoscopy was performed approximately one week later. It demonstrated numerous vascular ectasias diffusely distributed throughout the small bowel. Because of their extensive and multifocal distribution, an exact lesion count could not be reliably determined. The lesions appeared as flat erythematous vascular ectasias compatible with small-bowel angiodysplasia (Figure 1).

**Figure 2:**
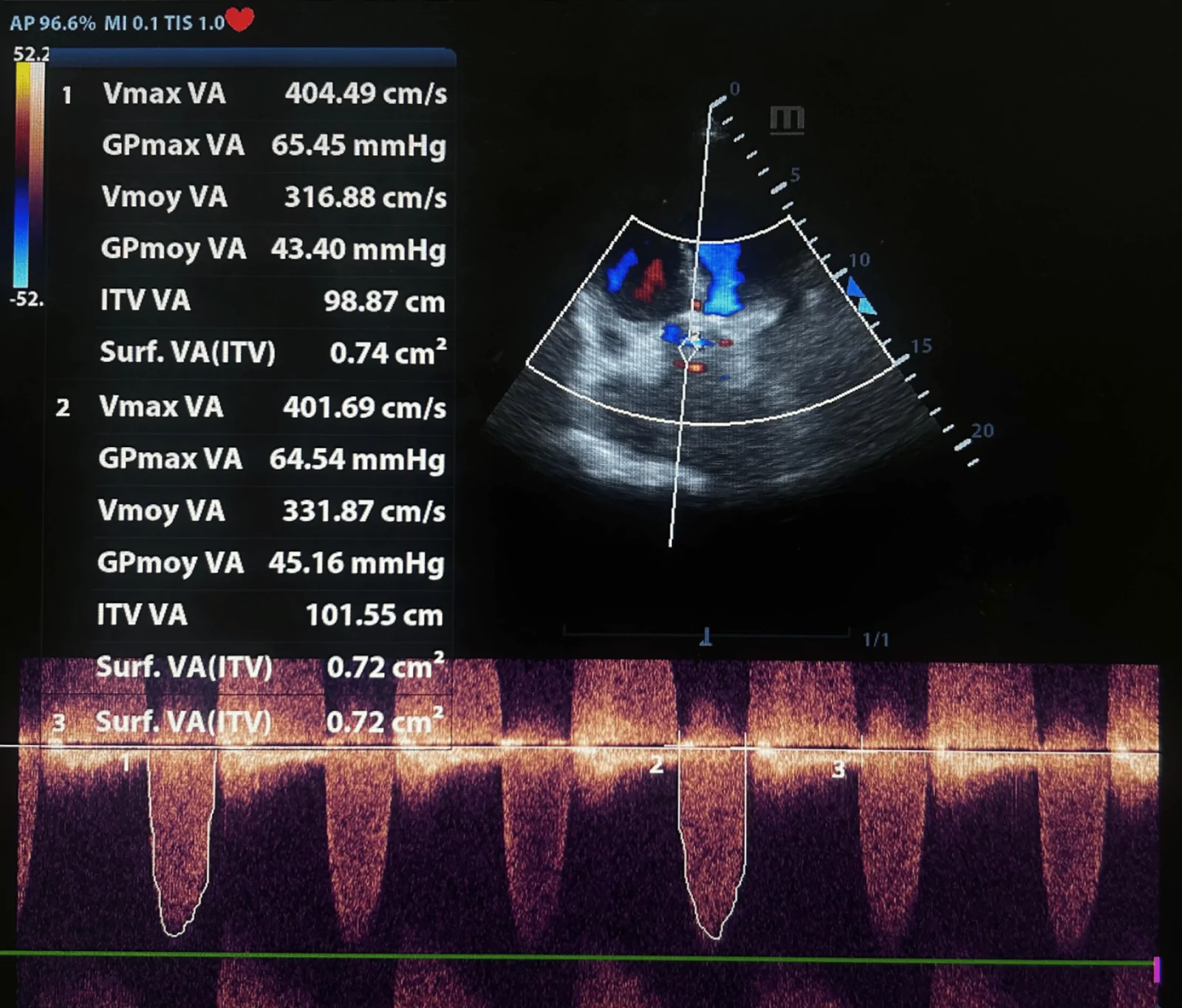
Echocardiographic assessment of aortic stenosis in Case 1. Continuous-wave Doppler echocardiography demonstrating severe aortic stenosis. Repeated measurements showed peak aortic jet velocities of 4.02-4.04 m/s, mean transvalvular gradients of 43.40-45.16 mmHg, and calculated aortic valve areas of 0.72-0.74 cm².

Transthoracic echocardiography confirmed severe calcific aortic stenosis. Repeated Doppler measurements showed a calculated aortic valve area of 0.72-0.74 cm², a mean transvalvular gradient of 43.40-45.16 mmHg, and a peak aortic jet velocity of 4.02-4.04 m/s (Figure 2). Moderate aortic regurgitation was also present. Left ventricular systolic function was preserved, with an ejection fraction of 55%.

Von Willebrand factor testing demonstrated an antigen level of 92% and an activity level of 38%, resulting in an activity-to-antigen ratio of 0.41. High-molecular-weight multimer analysis was not available at our center. Nevertheless, the disproportionately reduced activity, severe aortic stenosis, and recurrent gastrointestinal bleeding from small-bowel vascular lesions supported the diagnosis of acquired von Willebrand syndrome.

Device-assisted enteroscopy was not available at our center, which precluded an attempt at enteroscopic treatment of the numerous diffusely distributed small-bowel lesions. During the clinical course, the patient received one unit of packed red blood cells. Despite transfusion and supportive management, gastrointestinal bleeding persisted and resulted in progressive hemodynamic deterioration and hemorrhagic shock. Aortic valve replacement was considered necessary; however, the patient's clinical deterioration precluded definitive cardiac intervention. The patient ultimately died from hemorrhagic shock before valve replacement could be performed.

Case 2

A 70-year-old male was admitted for recurrent hematochezia that had been evolving for approximately two months, associated with chronic anemia. His medical history was significant for severe calcific aortic stenosis under regular cardiology follow-up. He was not receiving anticoagulant or antiplatelet therapy.

At presentation, the patient was hemodynamically stable. Cardiovascular examination revealed a systolic ejection murmur compatible with aortic stenosis, without clinical signs of heart failure. Laboratory evaluation demonstrated moderate anemia, with a hemoglobin level of 9 g/dL. Inflammatory markers were not elevated, and the platelet count and renal function were within reference ranges.

**Figure 3:**
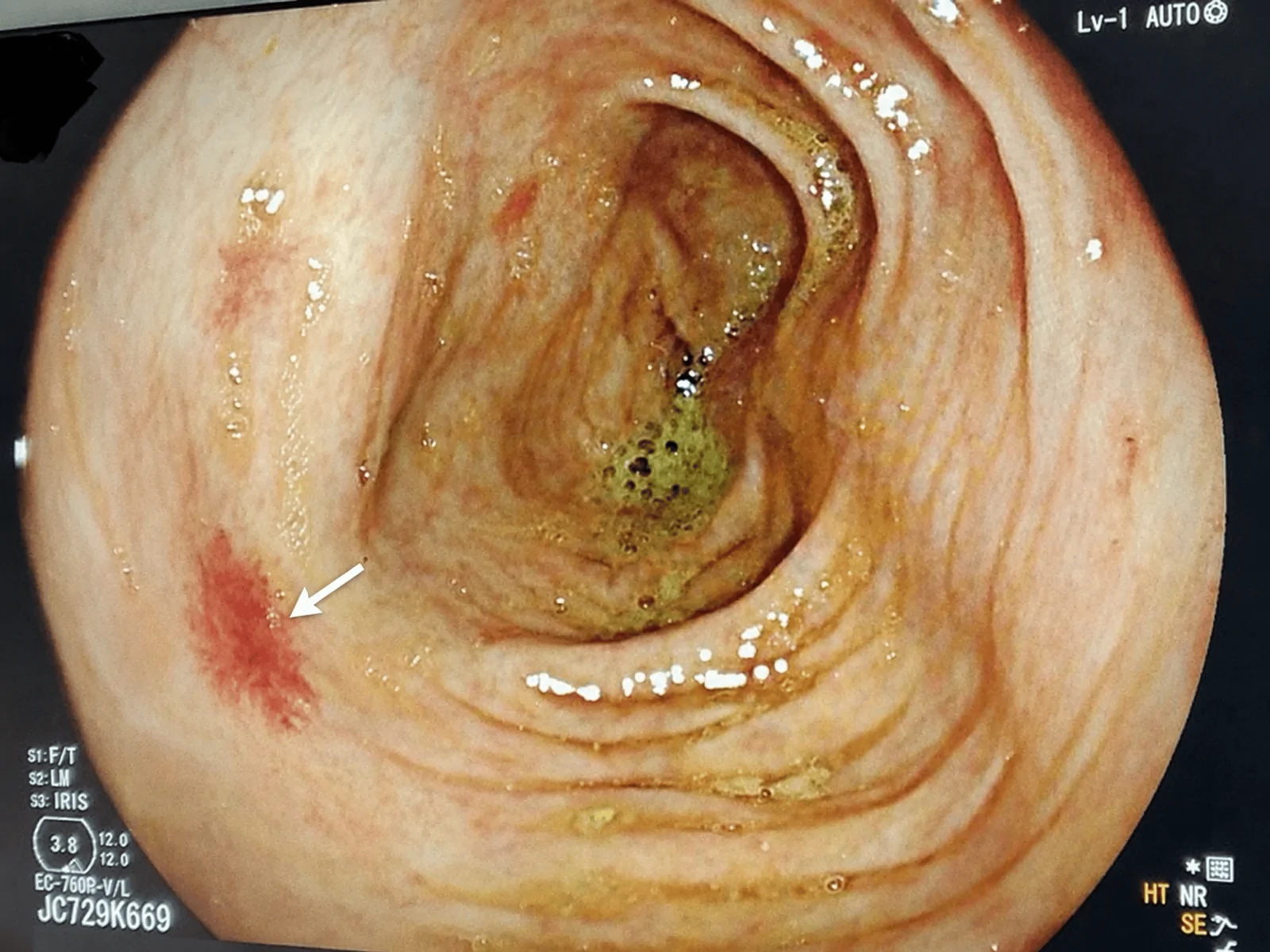
Right-colonic angiodysplasia in Case 2. Colonoscopy showing a localized vascular ectasia consistent with angiodysplasia in the right colon. The white arrow indicates the lesion, which was successfully treated with argon plasma coagulation.

Colonoscopy identified a localized angiodysplasia in the right colon, characterized by a flat erythematous vascular ectasia (Figure 3). Endoscopic treatment using argon plasma coagulation (APC) was successfully performed, resulting in immediate bleeding control.

**Figure 4:**
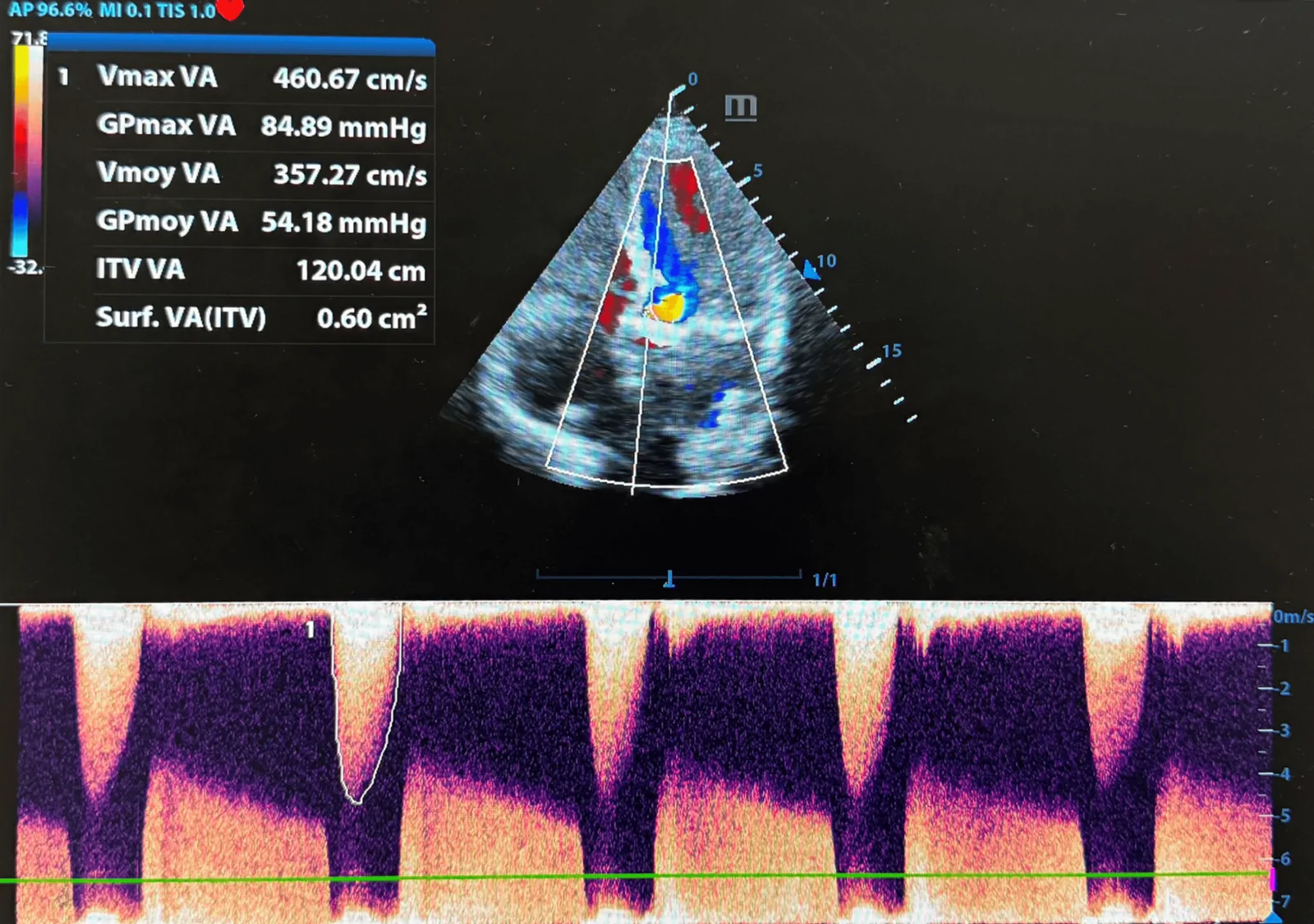
Echocardiographic assessment of aortic stenosis in Case 2. Continuous-wave Doppler echocardiography demonstrating severe aortic stenosis, with a peak aortic jet velocity of 4.61 m/s, a mean transvalvular gradient of 54.18 mmHg, and a calculated aortic valve area of 0.60 cm².

Transthoracic echocardiography demonstrated severe calcific aortic stenosis, with a calculated aortic valve area of 0.60 cm², a mean transvalvular gradient of 54.18 mmHg, and a peak aortic jet velocity of 4.61 m/s (Figure 4). Left ventricular systolic function was preserved, with an ejection fraction of 65%.

Von Willebrand factor antigen was 105%, whereas vWF activity was 48%, resulting in an activity-to-antigen ratio of 0.46. Multimer analysis was not available. These biological findings, in combination with severe aortic stenosis and bleeding from gastrointestinal angiodysplasia, supported the diagnosis of acquired von Willebrand syndrome.

After successful endoscopic hemostasis and correction of anemia, the patient was referred for definitive aortic valve intervention. No recurrent gastrointestinal bleeding was documented during one year of follow-up after APC. However, completion of the planned valve intervention could not be confirmed from the available records. Therefore, the absence of recurrent bleeding cannot be attributed to valve correction.

The principal clinical, endoscopic, cardiac, therapeutic, and outcome differences between the two patients, together with the relevant reference ranges or diagnostic thresholds, are summarized in Table 1.

**Table 1:** Comparison of the two patients with Heyde syndrome. Abbreviations: APC, argon plasma coagulation; AS, aortic stenosis; GI, gastrointestinal; vWF, von Willebrand factor. Laboratory reference intervals vary according to the assay and institution. Echocardiographic values are presented with commonly used diagnostic thresholds for severe aortic stenosis. * Exact numerical platelet and C-reactive protein values for Case 2 were not available in the archived record; they were documented as within the reference range and not elevated, respectively.

Characteristic	Case 1	Case 2	Reference range or diagnostic threshold
Age and sex	72-year-old male	70-year-old male	Not applicable
Duration of GI bleeding before presentation	Approximately 1 month	Approximately 2 months	Not applicable
GI presentation	Recurrent melena	Recurrent hematochezia	Not applicable
Hemoglobin	8.3 g/dL	9 g/dL	Approximately 13-17 g/dL in adult men; laboratory-dependent
Platelet count	112,000/mm³	Within reference range*	Approximately 150,000-400,000/mm³; laboratory-dependent
C-reactive protein	157 mg/L	Not elevated*	Usually <5 mg/L; laboratory-dependent
Initial endoscopic evaluation	Upper endoscopy and colonoscopy negative	Colonoscopy identified lesion	Not applicable
Interval to capsule endoscopy	Approximately 1 week after conventional endoscopy	Not applicable	Not applicable
Angiodysplasia location	Numerous diffuse small-bowel lesions	Localized right-colonic lesion	Not applicable
Lesion burden	Numerous; exact count not reliably determined	Localized lesion	Not applicable
Diagnostic modality	Capsule endoscopy	Colonoscopy	Not applicable
Endoscopic accessibility	Device-assisted enteroscopy unavailable at our center	Accessible by conventional colonoscopy	Not applicable
Endoscopic treatment	Not performed	Argon plasma coagulation	Not applicable
Packed red blood cells	1 unit	-	Not applicable
Aortic valve area	0.72-0.74 cm²	0.60 cm²	Severe AS: ≤1.0 cm²
Mean transvalvular gradient	43.40-45.16 mmHg	54.18 mmHg	Severe AS: ≥40 mmHg
Peak aortic jet velocity	4.02-4.04 m/s	4.61 m/s	Severe AS: ≥4.0 m/s
Left ventricular ejection fraction	55%	65%	Preserved in adult men: approximately ≥52%
vWF antigen	92%	105%	Commonly 50-150%; assay-dependent
vWF activity	38%	48%	Commonly 50-150%; assay-dependent
vWF activity-to-antigen ratio	0.41	0.46	Usually ≥0.7; assay-dependent
Clinical course	Persistent bleeding progressing to hemorrhagic shock	Immediate bleeding control after APC	Not applicable
Valve management	Planned but not performed	Referred for valve intervention; completion not confirmed	Not applicable
Outcome	Death from hemorrhagic shock before definitive valve treatment	No recurrent GI bleeding during one-year follow-up after APC	Not applicable

## Discussion

These two cases illustrate the heterogeneous clinical spectrum of Heyde syndrome. Both patients had severe calcific aortic stenosis, gastrointestinal bleeding from angiodysplasia, and biological findings supporting acquired von Willebrand syndrome. However, their immediate therapeutic options and clinical courses differed markedly. Case 1 had numerous angiodysplastic lesions diffusely distributed throughout the small bowel, whereas case 2 had a localized right-colonic angiodysplasia accessible to conventional colonoscopic treatment.

In severe aortic stenosis, increased shear stress across the narrowed valve promotes proteolytic degradation of high-molecular-weight von Willebrand factor multimers. The resulting acquired type 2A-like von Willebrand syndrome impairs platelet adhesion under high-shear conditions and increases susceptibility to gastrointestinal bleeding [3,4]. Angiodysplasias provide the structural bleeding substrate, while the associated hemostatic abnormality may increase the frequency and severity of hemorrhage [5-7].

Von Willebrand factor multimer analysis provides direct evidence of the selective loss of high-molecular-weight multimers; however, this test was not available at our center. In both patients, vWF activity was disproportionately reduced relative to antigen concentration, resulting in activity-to-antigen ratios of 0.41 and 0.46, respectively. These findings were interpreted in conjunction with severe aortic stenosis and gastrointestinal bleeding from angiodysplasia rather than as isolated laboratory confirmation of acquired von Willebrand syndrome [3,4].

In case 1, upper gastrointestinal endoscopy and colonoscopy failed to identify the bleeding source. Capsule endoscopy performed approximately one week later demonstrated numerous vascular ectasias diffusely distributed throughout the small bowel. Because of their extensive and multifocal distribution, an exact lesion count could not be reliably determined. Capsule endoscopy is an established investigation for suspected small-bowel bleeding and is particularly useful for detecting vascular lesions when conventional endoscopy is nondiagnostic [9-11]. Device-assisted enteroscopy can provide both diagnostic and therapeutic access to small-bowel lesions [10,11]; however, it was not available at our center. This resource limitation prevented an attempt at enteroscopic treatment of the numerous diffuse lesions.

In contrast, case 2 had a localized angiodysplasia in the right colon that was accessible to conventional colonoscopic treatment. APC is commonly used for the endoscopic treatment of gastrointestinal angiodysplasias and can provide effective hemostasis for accessible lesions [12]. In this patient, APC resulted in immediate bleeding control, and no recurrent gastrointestinal bleeding was documented during one year of follow-up. However, although the patient was subsequently referred for definitive aortic valve intervention, completion of surgical or transcatheter valve replacement could not be confirmed from the available records. Consequently, the favorable bleeding outcome cannot be attributed to valve correction, and the relative contribution of APC and the subsequent natural clinical course remains uncertain.

Endoscopic treatment may control an accessible bleeding lesion but does not correct the underlying hemodynamic mechanism of Heyde syndrome. Surgical aortic valve replacement and transcatheter aortic valve implantation reduce pathological shear stress and may restore the distribution of high-molecular-weight vWF multimers [13-15]. Correction of the valve abnormality is therefore considered central to definitive management and has been associated with a reduction in recurrent gastrointestinal bleeding. Early multidisciplinary management is particularly important in patients in whom recurrent bleeding complicates severe aortic stenosis [16]. In selected patients who are not immediately suitable for definitive valve replacement, temporary or bridging cardiovascular strategies may also be considered according to the clinical situation and local expertise [16].

Therapeutic options for recurrent or refractory angiodysplasia-related bleeding also extend beyond endoscopic treatment. Randomized evidence has supported the use of thalidomide and long-acting octreotide in selected patients with recurrent angiodysplasia-related bleeding [17,18]. Supportive treatment may include iron replacement and red blood cell transfusion when clinically indicated. Other hemostatic approaches, including desmopressin or vWF concentrate, may be considered in selected circumstances involving acquired von Willebrand syndrome, although such measures do not correct the underlying valvular shear-stress mechanism. In case 1, device-assisted enteroscopy was not available, and persistent bleeding continued despite supportive management.

The clinical course of case 1 was particularly severe. The patient received one unit of packed red blood cells, but gastrointestinal bleeding persisted and progressed to hemodynamic deterioration and hemorrhagic shock, which was the documented proximate cause of death. Definitive aortic valve intervention could not be performed before this deterioration. Nevertheless, the marked elevation in C-reactive protein to 157 mg/L and thrombocytopenia to 112,000/mm³ cannot be explained solely by angiodysplastic bleeding. These abnormalities may indicate an additional inflammatory or systemic process and represent an important limitation when interpreting the fatal outcome. Therefore, death should not be considered attributable solely to lesion distribution or lack of endoscopic accessibility.

The different outcomes of these two patients should consequently be interpreted cautiously. A two-patient case report cannot establish lesion distribution or therapeutic accessibility as independent prognostic factors. The numerous diffuse small-bowel lesions in case 1 limited available endoscopic therapeutic options, particularly because device-assisted enteroscopy was unavailable, whereas the localized right-colonic lesion in case 2 was immediately accessible to APC. However, case 1 also had thrombocytopenia, marked systemic inflammation, progressive hemorrhagic instability, and inability to undergo definitive valve treatment. In case 2, definitive valve intervention could not be confirmed. Thus, lesion localization and endoscopic accessibility may influence immediate bleeding management options, but the overall clinical outcome is likely multifactorial.

These cases also emphasize the importance of close collaboration between gastroenterology and cardiology teams. Gastrointestinal bleeding and anemia should be identified and stabilized while definitive management of severe aortic stenosis is considered. At the same time, unnecessary delay in valve intervention should be avoided when the patient's clinical condition permits. Multidisciplinary coordination may help balance control of gastrointestinal bleeding, correction of anemia, timing of cardiac intervention, and peri- and post-procedural antithrombotic considerations [16].

## Conclusions

Heyde syndrome should be considered in patients with severe aortic stenosis who present with recurrent or unexplained gastrointestinal bleeding. When upper gastrointestinal endoscopy and colonoscopy are nondiagnostic, capsule endoscopy may identify otherwise occult small-bowel vascular lesions. Endoscopic treatment can provide effective hemostasis for accessible angiodysplasias, whereas diffuse small-bowel disease may present greater therapeutic challenges, particularly when device-assisted enteroscopy is unavailable. Correction of the aortic valve abnormality remains central to definitive management. However, clinical outcome is multifactorial, and lesion distribution, endoscopic accessibility, systemic clinical status, and the feasibility and timing of valve intervention may all influence the clinical course. Early collaboration between gastroenterology and cardiology teams is important to control gastrointestinal bleeding and correct anemia while avoiding unnecessary delay in definitive cardiac treatment.
